# Immune Regulatory Mediators in Plasma from Patients With Acute Decompensation Are Associated With 3-Month Mortality

**DOI:** 10.1016/j.cgh.2019.08.036

**Published:** 2020-05

**Authors:** Natalia Becares, Suvi Härmälä, Louise China, Romain A. Colas, Alexander A. Maini, Kate Bennet, Simon S. Skene, Zainib Shabir, Jesmond Dalli, Alastair O’Brien

**Affiliations:** ∗ Centre for Clinical Pharmacology and Therapeutics, Division of Medicine, University College London, London, United Kingdom; ‡ Institute of Health Informatics, University College London, London, United Kingdom; § Lipid Mediator Unit, Center for Biochemical Pharmacology, William Harvey Research Institute, Barts and The London School of Medicine, Queen Mary University of London, Charterhouse Square, London, United Kingdom; ‖ Comprehensive Clinical Trials Unit, University College London, London, United Kingdom

**Keywords:** TNF, MDM, Death, Immune Response, ACLF, acute-on-chronic liver failure, AD, acute decompensation, ATTIRE, Albumin to Prevent Infection in Chronic Liver Failure, CAID, cirrhosis-associated immune dysfunction, CRP, C-reactive protein, DPA, docosapentaenoic acid, HAS, human albumin solution, IL, interleukin, IV, intravenous, LBP, lipopolysaccharide-binding protein, LM, lipid mediator, LPS, lipopolysaccharide, MDM, monocyte-derived macrophage, MELD, Model for End-Stage Liver Disease, PBMC, peripheral blood mononuclear cell, PGE_2_, prostaglandin E_2_, RCT, randomized controlled trial, SPMs, specialized pro-resolving mediators, TNF, tumor necrosis factor, WCC, white cell count

## Abstract

**Background & Aims:**

Infection is a common cause of death in patients with cirrhosis. We investigated the association between the innate immune response and death within 3 months of hospitalization.

**Methods:**

Plasma samples were collected on days 1, 5, 10, and 15 from participants recruited into the albumin to prevent infection in chronic liver failure feasibility study. Patients with acute decompensated cirrhosis were given albumin infusions at 10 hospitals in the United Kingdom. Data were obtained from 45 survivors and 27 non-survivors. We incubated monocyte-derived macrophages from healthy individuals with patients’ plasma samples and measured activation following lipopolysaccharide administration, determined by secretion of tumor necrosis factor and soluble mediators of inflammation. Each analysis included samples from 4 to 14 patients.

**Results:**

Plasma samples from survivors vs non-survivors had different inflammatory profiles. Levels of prostaglandin E2 were high at times of patient hospitalization and decreased with albumin infusions. Increased levels of interleukin 4 (IL4) in plasma collected at day 5 of treatment were associated with survival at 3 months. Incubation of monocyte-derived macrophages with day 5 plasma from survivors, pre-incubated with a neutralizing antibody against IL4, caused a significant increase in tumor necrosis factor production to the level of non-survivor plasma. Although baseline characteristics were similar, non-survivors had higher white cell counts and levels of C-reactive protein and renal dysfunction.

**Conclusions:**

We identified profiles of inflammatory markers in plasma that are associated with 3-month mortality in patients with acute decompensated cirrhosis given albumin. Increases in prostaglandin E2 might promote inflammation within the first few days after hospitalization, and increased levels of plasma IL4 at day 5 are associated with increased survival. Clinicaltrialsregister.eu: EudraCT 2014-002300-24

**What You Need to Know**

**Background**

Infection is a common cause of death in patients with cirrhosis. We investigated the association between the innate immune response and death within 3 months of hospitalization.

**Findings**

In an analysis of plasma samples from patients given albumin infusions for acute decompensated cirrhosis or acute on chronic liver failure, we found differences in levels of cytokines in patients who survived 30 days vs patients who did not. Plasma from survivors had increased levels of interleukin 4, which reduced activation of monocyte-derived macrophages. Plasma levels of prostaglandin E_2_ decreased with albumin treatment.

**Implications for patient care**

Differences in plasma levels of cytokines, such as prostaglandin E_2_ and interleukin 4, might affect response to infections or treatment in patients with acute decompensated cirrhosis or acute on chronic liver failure.

The most severe clinical presentation of liver cirrhosis is acute decompensation (AD) or acute-on-chronic-liver failure (ACLF).^1^ Patients are highly prone to bacterial infection caused by immune dysregulation^2^ termed *cirrhosis-associated immune dysfunction* (CAID).^2^ CAID causes a paradoxical phenotype in ACLF that combines exaggerated systemic inflammation with immune suppression. Potential immune restorative therapies should aim to improve immune function without worsening systemic inflammation; however, despite detailed work describing the ACLF phenotype^3^^,^^4^ and its high clinical relevance, there are no licensed treatments to improve immune dysfunction.

We previously identified prostaglandin E_2_ (PGE_2_) as a potential causative immune suppressive molecule.^5^^,^^6^ Albumin has been reported to bind and catalyze PGE_2_ inactivation,^7^ and we found that as albumin levels decreased in AD/ACLF, PGE_2_ may be more bioavailable and injurious. We therefore proposed transfusing 20% human albumin solution (HAS) to antagonise the effects of PGE_2_^6^ and prevent infection in our randomized controlled trial (RCT), ATTIRE (Albumin to Prevent Infection in Chronic Liver Failure). In the single-arm ATTIRE feasibility study of 79 patients at 10 sites, we demonstrated that 20% HAS infusions restored serum albumin levels to >30 g/dL and improved ex vivo immune function in AD/ACLF patients by day 3 of study participation through antagonism of PGE_2_.^6^^,^^8^ However, this study included samples from only the first few days of admission and were not linked with clinical outcome.

We therefore performed this follow-up study examining the inflammatory response throughout admission in albumin-treated patients and linked this to outcome. We selected mortality at 3 months after recruitment as our primary clinical outcome to study whether the inflammatory response throughout admission differed between survivors and non-survivors and potential underlying molecular mechanisms.

Our study suggests that survivors and non-survivors exhibited distinct temporal profiles in immune function that corresponded with changes in white cell count (WCC), and we propose a novel role for interleukin (IL) 4 in this process.

## Methods

### Patient Studies

Patients were recruited as part of the ATTIRE feasibility study; all were treated with daily intravenous (IV) 20% HAS if serum albumin <30 g/L during the trial treatment period (up to 14 days after recruitment). All patients admitted to hospital with AD/severe worsening of liver cirrhosis complications, aged >18 years, serum albumin <30 g/L, predicted hospital admission by attending clinicians more than 5 days, and for full active management at admission were eligible. Patients were recruited within 72 hours of hospitalization; full criteria are described elsewhere.^8^^,^^9^ We sought written informed patient consent from patients or representatives if they lacked capacity. Research ethical approval was granted by London-Brent research ethics committee (ref: 15/LO/0104). Plasma samples were randomly selected corresponding to days 1 (pre-treatment), 5, 10, and 15 (end of trial). Survivor and non-survivor groups were divided a priori on the basis of death during 3-month follow-up at local National Health Service sites. Data were obtained from a maximum 45 survivors and 27 non-survivors at baseline. Experimental studies were performed on samples available, with n values in figure legends.

The trial is registered with European Medicines Agency (EudraCT 2014-002300-24) and adopted by National Institute for Health Research (ISRCTN14174793). All authors had access to the study data and reviewed and approved the final manuscript.

Laboratory analysis is described in Supplementary Methods. For multiple comparisons, significance was assessed by one-way analysis of variance, followed by Bonferroni adjusted pairwise *t* tests. WCC and C-reactive protein (CRP) followed an approximately log-normal distribution each day, and values were therefore transformed (ln, natural logarithm) for statistical analyses. Numbers of tests were adjusted for the number of trial days. Differences in mean values between survivors and non-survivors were compared by using two-tailed *t* test, allowing for unequal variances with Bonferroni correction.

## Results

### Distinct Plasma-Mediated Inflammatory Response Phenotypes During Hospitalization in Albumin-Treated Patients Differentiated Between Survivors and Non-Survivors

**Figure 1:**
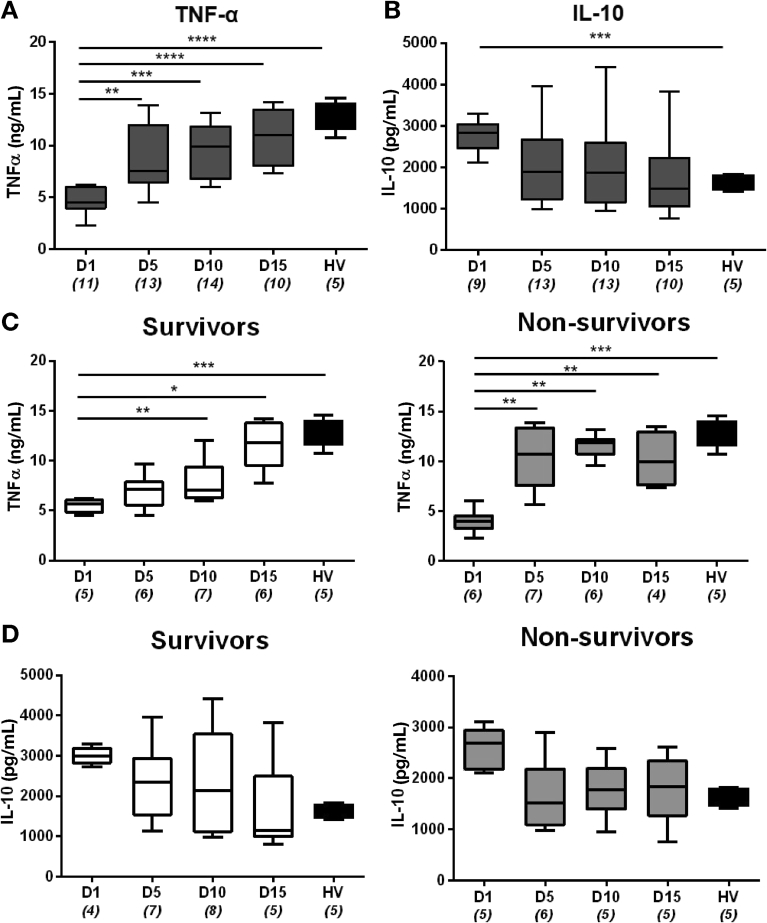
Distinct immune restoration phenotype associated with survival develops over time. Levels of TNF-α (*A* and *C*) and IL10 (*B* and *D*) secreted by MDMs were quantified by enzyme-linked immunosorbent assay. Cells were sensitized with plasma from ATTIRE survivors, non-survivors, and healthy volunteers (HV) for 30 minutes and stimulated with 100 ng/mL lipopolysaccharide for 4 (*A* and *C*) and 24 (*B* and *D*) hours. *Boxes* represent median with upper and lower quartiles. *Bars* represent minimum and maximum observations. Numbers of samples used for each day are written in parentheses. *P < .05, **P < .01, ***P < .001. Significance determined by using one-way analysis of variance and Bonferroni adjusted pairwise *t* tests. ATTIRE, Albumin to Prevent Infection in Chronic Liver Failure; IL, interleukin; MDM, monocyte-derived macrophage; TNF, tumor necrosis factor.

We investigated the temporal profile of inflammatory responses in participants throughout the trial period by examining the effect of plasma from days 1, 5, 10, and 15 on healthy monocyte-derived macrophages (MDMs).^5^^,^^6^ MDMs were incubated with plasma from AD/ACLF patients or healthy volunteers and subsequent activation in response to lipopolysaccharide (LPS) quantified by assessing levels of secreted tumor necrosis factor (TNF)-α. As previously,^6^ plasma from AD/ACLF patients at day 1 reduced MDM TNF-α production compared with healthy volunteer plasma (Figure 1*A*). This immune suppressive effect waned during hospitalization, and after targeted HAS infusions, it reached healthy volunteer levels by end of trial treatment period (day 15) (Figure 1*A*). MDM production of the anti-inflammatory cytokine IL10 at 24 hours after stimulation mirrored this pattern with levels higher at day 1 and returning to those produced by cells incubated with healthy plasma by day 15 (Figure 1*B*).

When samples were divided into survivor and non-survivors, this observed pattern of immune activation differed between groups. MDMs sensitized with day 1 non-survivor plasma demonstrated a slightly lower production of TNF-α compared with survivors (Figure 1*C*). However, MDM TNF-α production in this group was rapidly and significantly restored to healthy levels by day 5 and maintained throughout the rest of the trial. In contrast, MDM TNF-α production after treatment with survivor plasma did not reach healthy plasma levels until day 15 of the trial (Figure 1*C*). Indeed, there was a 2.6-fold increase in TNF-α levels between day 1 and day 5 in cells incubated with non-survivor plasma (*P* < .001) compared with 1.26-fold increase in those treated with survivor plasma (*P* > .05). Neither patient group’s plasma elicited greater TNF-α production than healthy volunteer plasma by day 15 of trial (Figure 1*C*). These distinct patterns of inflammatory response between survivors and non-survivors were mirrored by MDM production of IL10 (Figure 1*B*). Survivor plasma elicited a gradual decrease in MDM IL10 production to reach the same level as healthy volunteer plasma by day 15, whereas non-survivor plasma elicited a more rapid fall in production by day 5, although differences did not reach significance (Figure 1*D*). These 2 inflammatory response phenotypes (rapid in non-survivors compared with gradual in survivors) appeared distinct by day 5 of the trial, marking this as a potential crucial time frame that determines outcome. We therefore focused further analyses between baseline (day 1) and day 5.

### Both Groups of Patients Demonstrated Similar Baseline Clinical Characteristics but Non-Survivors Developed Increased Serum White Cell Counts, C-Reactive Protein, and Renal Dysfunction

**Figure 2:**
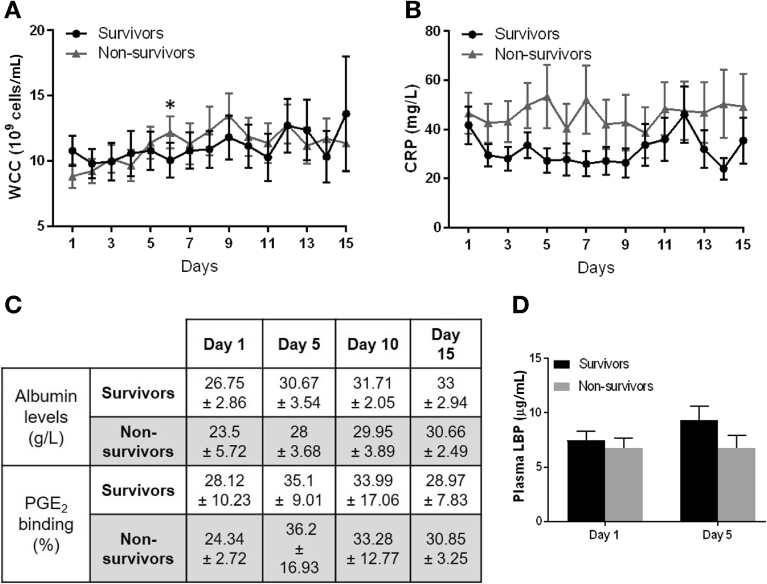
Plasma levels of albumin, LBP, and albumin-PGE_2_ binding capacity did not differentiate between groups. (*A* and *B*) Daily white cell counts (WCC) (*A*) and C-reactive protein (CRP) (*B*) in survivor (n = 10–38) and non-survivor (n = 6–22) groups during ATTIRE feasibility study. Data expressed as mean and standard error. (*C*) Levels of circulating albumin (g/L) (n = 5–7) and percentage of PGE_2_/3H-PGE_2_ bound to survivor and non-survivor patient plasma protein, days 1, 5, 10, and 15 using equilibrium dialysis (n = 3–5). (*D*) Plasma lipopolysaccharide-binding protein (LBP) by enzyme-linked immunosorbent assay in days 1 and 5 samples from survivors (n = 5–8) and non-survivors (n = 7–10). ATTIRE, Albumin to Prevent Infection in Chronic Liver Failure; PGE_2_, prostaglandin E_2_.

Overall baseline patient data have been presented previously in this cohort, with the majority having alcohol-related cirrhosis.^8^ Non-survivors displayed non-significantly increased Model for End-Stage Liver Disease (MELD) scores, ACLF scores, diagnosis of infection at baseline, and antibiotic prescription (Table 1). The number of days in trial, baseline WCC, and CRP were similar in both groups (Table 2). Serum creatinine was higher in non-survivors at baseline, and renal dysfunction developed solely in the non-survivor group (30.8%, Table 1). Because the differences in laboratory inflammatory response appeared during the first half of the trial, we compared the WCC and CRP values between groups during this period (up to day 7). On day 6 where the difference in WCC levels between groups reached its peak (Figure 2*A*), the absolute WCC levels were significantly higher in non-survivors (*P* = .029). Relative change from baseline WCC also significantly differed on day 6 between survivors and non-survivors (adjusted *P* = .033, corrected for 4 tests, raw *P* = .008) when values were compared. CRP followed a similar trend with higher levels in non-survivors, but differences in absolute levels did not reach statistical significance; however, the difference in relative change from baseline CRP significantly differed between groups on day 4 (adjusted *P* = .032, corrected for 2 tests, raw *P* = .016) (Figure 2*B*). Nosocomial infections developed in 10 of 26 non-survivors compared with 9 of 45 survivors after 48 hours of albumin treatment (*P* = .19, χ^2^ test).

**Table 1:** Patient Group Characteristics

	Survivors		Non-survivors	
Median age, *y*, range (n = number of patients with data)	54.6, 30–75 (45)		55.3, 24–81 (27)	
Male sex, n (*%*)	32/45 (71.1)		15/27 (55.5)	
Baseline median MELD score, IQR (n)	19.8, 16.0–23.1 (38)		23.6, 17.4–28.4 (20)	
Total HAS given, median, IQR (n)	850, 700–1500 (44)		1000, 400–1600 (26)	
Median days in trial, IQR (n)	14, 8–15 (44)		12, 4–15 (26)	
No. with infection diagnosed at recruitment (*%*)	16/45 (35.5)		12/26 (48)	
Prescribed antibiotics at recruitment, n (*%*)	22/45 (49)		17/26 (65)	
No. with new infection during trial after HAS treatment for >48 h (*%*)	9/45 (20)		10/26 (37)	
Renal dysfunction diagnosis during trial (*%*)	0/44 (0.0)		8/26 (30.8)	
Alcohol consumption as etiology (*%*)	43 (91)		27 (100)	
Active alcohol consumption at admission (*%*)	20 (43)		5 (19)	
ACLF scores, n (*%*)	0	35 (77.8)	0	17 (63)
	1	6 (13.3)	1	4 (14.8)
	2	3 (6.7)	2	3 (11.1)
	3	1 (2.2)	3	3 (11.1)

ACLF, acute-on-chronic liver failure; HAS, human albumin solution; IQR, interquartile range; MELD, Model for End-Stage Disease.

**Table 2:** Baseline Blood Tests of Survivors and Non-survivors

Median (IQR)	Survivors (n = 45)	Non-survivors (n = 27)
Albumin (*g/L*)	24.0 (21.5–26.5)	25.0 (22.0–27.0)
Bilirubin (*μmol/L*)	98.5 (50.5–244.5)	107.0 (64.0–275.0)
White cell count (*10*^*6*^*cells/mL*)	9.1 (6.2–12.6)	8.5 (5.7–11.5)
C-reactive protein (*mg/L*)	29.5 (11.0–55.0)	37.5 (16.0–67.0)
Creatinine (*mmol/L*)	65.0 (52.0–81.0)	80.0 (63.0–118.0)
International normalized ratio	1.7 (1.4–1.8)	1.7 (1.4–2.2)
Temperature (*°C*)	36.8 (36.5–37.5)	36.5 (36.1–36.8)

IQR, interquartile range.

### Plasma Levels of Albumin, Lipopolysaccharide-Binding Protein, and Albumin-Prostaglandin E_2_ Binding Capacity During Hospitalization Did Not Differ Between Survivors and Non-Survivors

Volume of HAS administered during the trial did not differ between survivor and non-survivor groups (Table 1), and levels of circulating albumin and its binding capacity to PGE_2_ improved similarly in both groups throughout the trial (Figure 2*C*). Levels of plasma lipopolysaccharide-binding protein (LPB), used as a marker of endotoxin presence,^10^ did not differ between groups (Figure 2*D*).

### Plasma Lipid Mediator Profiles at Days 1, 5, 10, and 15 Differed Between Survivors and Non-Survivors

**Figure 3:**
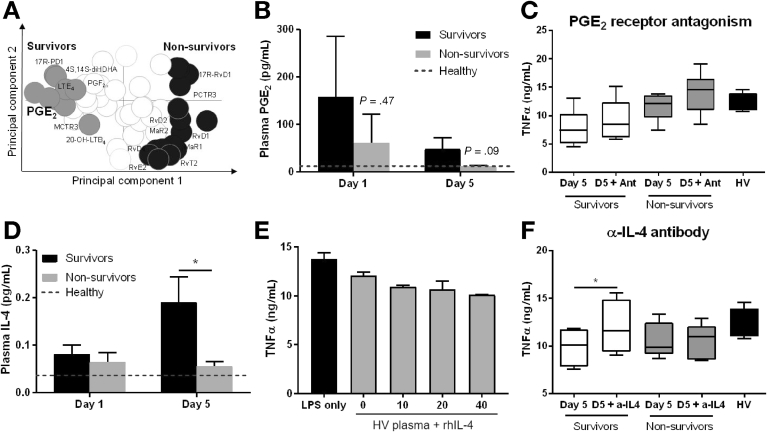
Plasma molecule study reveals differential IL4 levels that associate with survival. (*A*) Two-dimensional loading plot of plasma samples at day 5 (*C, D*). Survivors (n = 7), non-survivors (n = 8). (*B*) Levels of PGE_2_ in human plasma from acute decompensation patients collected at indicated intervals. Results expressed as pg/mL, mean ± standard error of the mean. *Dotted line* represents mean levels from healthy volunteer plasma (n = 5). *P* values correspond to unpaired *t* test (between survivors and non-survivors at days 1 and 5). (*C*) Levels of secreted TNF-α by MDMs quantified by ELISA. Cells were pre-treated with 50 μmol/L AH6809 (EP1-3 antagonist) and 10 μmol/L MF498 (EP4 antagonist) and sensitized with day 5 plasma from ATTIRE survivors and non-survivors and healthy volunteers (HV) for 30 minutes and stimulated with 100 ng/mL LPS for 4 hours. Data represent median with 25th/75th percentiles (n = 4–6). *Bars* represent minimum and maximum observations. (*D*) Plasma IL4 levels on day 1 and day 5 samples from survivors (n = 8–9) and non-survivors (n = 7–8). Results expressed as pg/mL, mean ± standard error of the mean. *P < .05 determined by Student *t* test. *Dotted line* represents mean levels from healthy volunteer plasma (n = 5). (*E* and *F*) Levels of secreted TNF-α by MDMs quantified by ELISA. Cells were sensitized with healthy volunteer plasma supplemented with several concentrations of recombinant human (rh) IL4 (*E*) or day 5 plasma from survivors and non-survivors incubated with neutralizing anti-IL4 antibody (*F*) for 30 minutes and stimulated with 100 ng/ml LPS for 4 hours (n = 4–5). *P < .05 determined by paired *t* test. ATTIRE, Albumin to Prevent Infection in Chronic Liver Failure; ELISA, enzyme-linked immunosorbent assay; IL, interleukin; LPS, lipopolysaccharide; MDM, monocyte-derived macrophage; TNF, tumor necrosis factor.

**Supplementary Figure 1:**
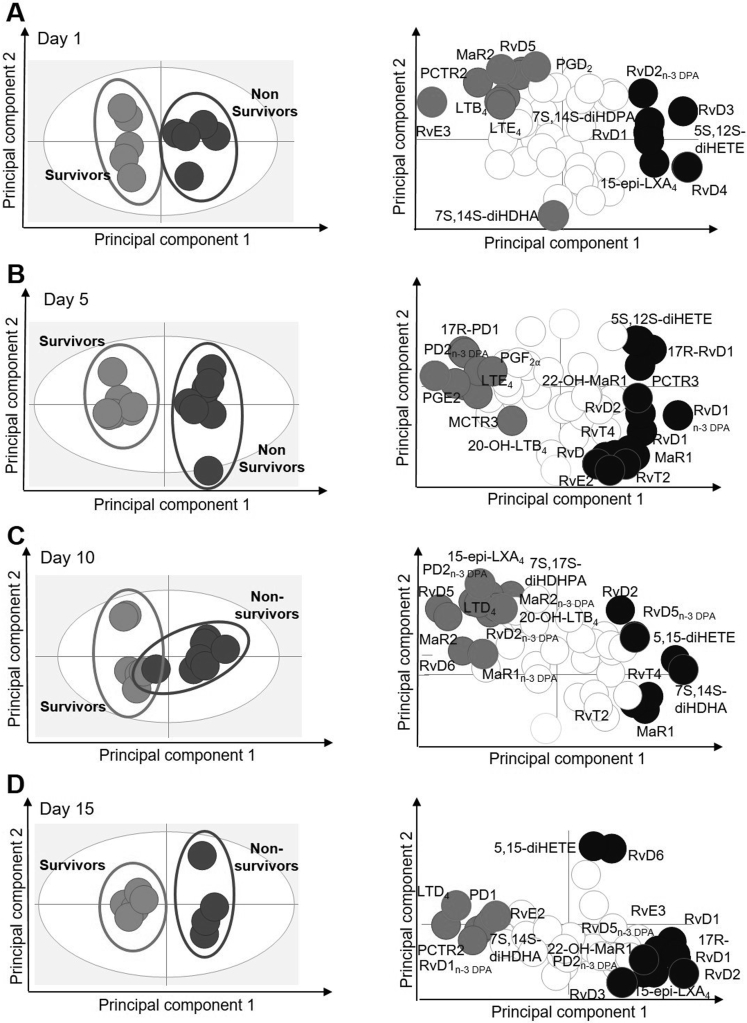
(*A–C*) Two-dimensional score plot (*left*) and loading plot (*right*) of samples at day 1 (*A*), day 5 (*B*), day 10 (*C*), and day 15 (*D*). *Gray ellipse* in the score plot denotes 95% confidence interval regions. Day 1: survivors n = 5, non-survivors n = 5; day 10: survivors n = 6, non-survivors n = 7; day 15: survivors n = 5, non-survivors = 4.

Lipid mediator (LM) analysis revealed differing trends between inflammation-initiating and resolution pathways in survivors and non-survivors according to day of sampling. We observed differences between docosahexaenoic acid–derived pro-resolving LMs (SPMs): D-series resolvins (RvD1, RvD2, RvD3, RvD4, RvD5, RvD6, 17R-RvD1, and 17R-RvD3), protectins (PD1 and 17R-PD1), and maresins (MaR1 and MaR2); n-3 docosapentaenoic acid (DPA)-derived SPMs: resolvins (RvT1, RvT3, RvT4, RvD1n-3 DPA, RvD2n-3 DPA, and RvD5n-3 DPA), protectins (PD1n-3 DPA), and maresins (MaR1n-3 DPA); EPA-derived SPMs: E-series resolvins (RvE1, RvE2, and RvE3); and differences were observed in arachidonic acid–derived lipoxins (LXA4, LXB4, 15-epi-LXA4, and 15-epi-LXB4) (Figure 3*A*, Supplementary Figure 1).

Using orthogonal partial least square discriminant analysis, a regression model that identifies variables contributing to separation of experimental groups, we found LM profiles of survivors were distinct from non-survivors, demonstrated by divergent clustering of LM profiles for patients from each group for all intervals tested (Supplementary Figure 1). Assessment of the variable in importance scores, which identify the contribution of each mediator in the observed separation between each of the groups, demonstrated an increased concentration of several SPM mediators associated with non-survivors (Supplementary Figure 1). Members of the D-series resolvin family were linked with non-survivors, although increases in absolute amounts of these molecules did not reach statistical significance (Supplementary Table 1).

Plasma LM profiles demonstrated PGE_2_ concentrations were similarly elevated levels at day 1 (Figure 3*A* and *B*) (*P* = .47) and fell substantially by day 5 in both groups (*P* = .09). When cells incubated with day 5 plasma from both survivors and non-survivors were treated with pan-PGE_2_ receptor antagonists, there was a similar increase in MDM TNF-α production (1.2-fold change for both groups) (Figure 3*C*).

### Elevated Plasma Interleukin 4 Concentration at Day 5 Was Associated With 3-Month Survival and Was Able to Switch Plasma-Mediated Inflammatory Response

**Supplementary Figure 2:**
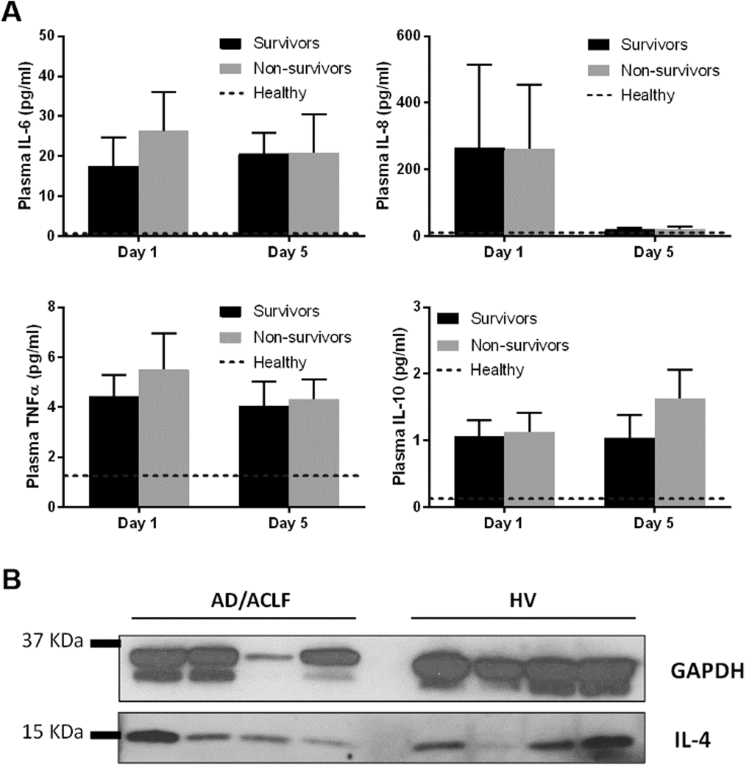
(*A*) Plasma cytokine levels on day 1 and day 5 samples from survivors (n = 8–9) and non-survivors (n = 7–8). Results are expressed as pg/mL, mean ± standard error of the mean. *Dotted line* represents mean levels on healthy volunteer plasma (n = 5). (*B*) IL4 was detected by immunoblotting on peripheral blood mononuclear cells of acutely decompensated (AD) patients and healthy volunteers (HV). Expression of GAPDH was used as input loading control. ACLF, acute-on-chronic liver failure; GAPDH, glyceraldehyde-3-phosphate dehydrogenase; IL, interleukin; TNF, tumor necrosis factor.

**Supplementary Figure 3:**
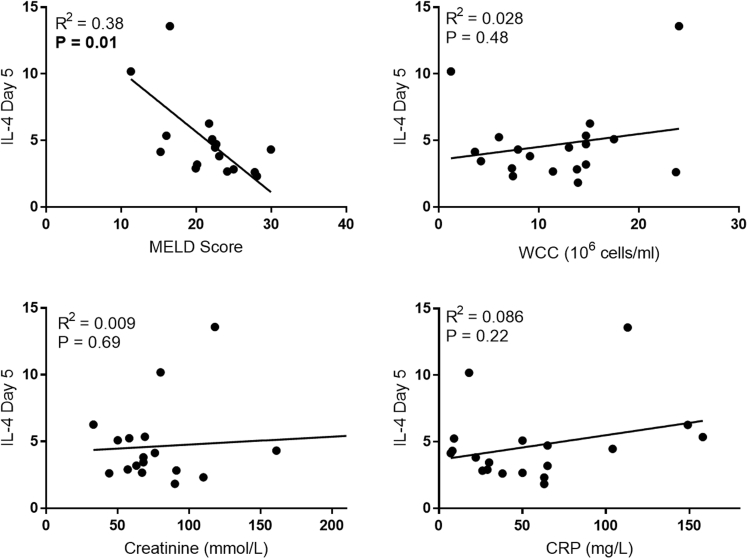
Day 5 plasma IL4 levels correlations with baseline clinical characteristics. Data represent product of Pearson correlation coefficient (n = 13–16). Best-fit linear slope is also included. CRP, C-reactive protein; IL, interleukin; MELD, Model for End-Stage Liver Disease; WCC, white cell count.

We measured 30 cytokines in survivor and non-survivor plasma from days 1, 5, 10, and 15 by multiplex analysis (Supplementary Table 2). No differences were observed between groups of the classic inflammatory cytokines linked to ACLF prognosis,^11^^,^^12^ such as TNF-α, IL6, IL8, and IL10 (Supplementary Figure 2*A*). A significant increase in IL4 was found in survivor day 5 plasma (Figure 3*D*), the period at which survivor and non-survivor plasma-mediated inflammatory response phenotypes appeared distinct. Western blot analysis revealed that peripheral blood mononuclear cells (PBMCs) from AD/ACLF patients contained detectable levels of IL4 protein (Supplementary Figure 2*B*). Subsequently, we demonstrated that MDMs incubated with healthy volunteer plasma that had been supplemented with increasing concentrations of recombinant IL4 caused decreased TNF-α production in response to LPS in a dose-dependent manner (Figure 3*E*). MDM cells treated with day 5 survivor plasma that had been preincubated with neutralizing anti-IL4 antibody (black and white squared box) significantly increased TNF-α production, and this reached the same level as seen in cells incubated with non-survivor plasma (light grey box) (Figure 3*F*). No effect was seen with neutralizing anti-IL4 antibody in experiments using non-survivor plasma (Figure 3*F*). Finally, we observed that IL4 plasma concentrations at day 5 correlated negatively with baseline MELD score (Supplementary Figure 3).

## Discussion

Immune function and inflammation in AD/ACLF patients have emerged as a critical area of research with the aim of developing treatments that improve mortality.^13^ Because of challenges associated with collecting primary cells from multiple sites, we used our ex vivo immune assay to investigate plasma-mediated immune responses and sought to determine whether results were associated with 3-month mortality. These data demonstrate that HAS infusions continued to restore the plasma-mediated inflammatory response as defined by macrophage TNF-α and IL10 production in AD/ACLF patients to healthy volunteer levels beyond day 3 as shown previously.^6^^,^^8^ This is consistent with recent data identifying changes in plasma IL10 reflecting disease severity and improving after albumin treatment.^4^^,^^14^ Survivors and non-survivors demonstrated distinct and unexpected plasma-mediated inflammatory response patterns during the trial, with non-survivors exhibiting a rapid increase of MDM activation to healthy levels that associated with an elevated WCC. Despite a significant overall effect on our immune assay, the HAS-PGE_2_ interaction did not appear to differentiate between these inflammatory phenotypes. Intriguingly, IL4 at day 5 was significantly up-regulated in survivors, and mechanistic analyses suggest this may also represent a potential therapeutic target. Finally, a failed resolution of inflammation LM phenotype was observed in non-survivors.

Our novel data support a mechanistic protective role for IL4. This cytokine has not been previously linked to CAID, although studies in humans and animal models support a protective role for elevated IL4 in sepsis^15^^,^^16^ and acute lung injury.^17^ We demonstrated that manipulation of IL4 switched immune restoration-like phenotypes between survival and non-survival and suggests IL4 agonism may represent a potential immune-restorative target in AD/ACLF patients who develop increased circulatory inflammatory markers during hospitalization.

Previous studies demonstrated an association between high circulatory inflammatory markers, eg, CRP,^18^^,^^19^ and inflammatory cytokines at baseline^12^^,^^20^^,^^21^ and increased mortality in AD/ACLF. In our cohort baseline clinical characteristics of the survivors and non-survivors were quite similar aside from serum creatinine and a non-significant increase in MELD score and infection rate, which may be due to the relatively small sample size. However, the development of an elevated WCC, CRP, and renal dysfunction during admission was significantly associated with 3-month mortality. Our work supports elevated PGE_2_ being crucial in this process early in admission and IL4 at later time points, although it is likely that other molecules are involved.

We previously showed that albumin treatment may alter plasma LM profiles in a small cohort of patients from the ATTIRE feasibility study.^6^ In this study principal component analysis revealed several families of SPMs that associated with good or poor outcome using samples taken at different time points before and after albumin infusions. This work remains promising, but greater numbers of patients will be required to identify specific LMs for potential drug development targets or to predict outcome or response to treatment. The observation that SPMs are also up-regulated in non-survivors indicates that although SPM biosynthetic pathways are active in these patients, production does not limit the unbridled inflammatory response. This could be due to delayed engagement of biosynthetic pathways, dysregulated receptor expression, or dysregulated downstream signaling pathways.

Many studies have demonstrated the potential immunomodulatory properties of albumin.^5^^,^^6^^,^^22^ However, differing results from recent well-conducted outpatient studies suggest patient response may not be uniform.^23^^,^^24^ All patients in this single-arm study were treated with IV HAS, which appeared to have a beneficial immune effect throughout hospitalization by antagonizing PGE_2_ effects. However, it is possible that the differing inflammatory responses observed associated with patients’ 3-month outcome may represent albumin treatment “successes” and “failures”. Further studies are required to determine whether PGE_2_ levels early in admission, IL4 levels at day 5, or a LM failure to resolve phenotype represent biomarkers or targets for a future precision medicine approach to improve treatment in these patients. This was a single-arm study, and we were unable to compare findings with non-treated patients.

Our immune assay was developed to investigate effects of plasma from AD/ACLF patients on macrophage function rather than primary cells, because it was not possible to collect fresh monocytes from multiple United Kingdom sites. However, our separate single site study has demonstrated an identical phenotype of reduced TNF-α production in fresh whole blood from AD/ACLF patients stimulated with LPS, supporting this approach.^25^ Furthermore, our laboratory assay was potentially able to differentiate between survivors and non-survivors, and mechanistic work has identified a potential novel mediator of CAID, suggesting that the approach has validity. The association of the inflammatory phenotype in those who died at 3 months with development of an elevated WCC and CRP strengthens the laboratory work and aids potential clinical approaches in the future. Our cohort predominantly included patients with alcohol-driven liver disease, and the laboratory analyses used samples only from patients with alcohol-related cirrhosis. Therefore, our findings may not be applicable to cohorts containing large numbers of nonalcoholic steatohepatitis or viral hepatitis patients. Moreover, we did not have sufficient samples to extend studies to compare between AD and ACLF. ATTIRE stage 2, a large-scale RCT at 30 sites that finishes in June 2019,^26^ will provide samples taken from patients treated with and without HAS at days 1, 5, and 10 to validate these findings and guide future immune restorative approaches in AD/ACLF. This will allow us to discern the potential effect of 20% HAS infusion on the establishment of different patterns of inflammatory responses observed and their relationship to survival.

We present novel data that describe CAID as a dynamic process and propose that the inflammatory response trajectory may represent the critical determinant of clinical outcome. Distinct plasma-mediated inflammatory responses, as defined by our laboratory assay and changes in WCC and CRP from baseline during the first week of hospitalization, were associated with organ dysfunction and death in albumin-treated advanced liver disease patients. Finally, our results show PGE_2_ and IL4 as pivotal mediators that underlie the inflammatory response in these patients at different time points during their hospital admission.

## Figures and Tables

**Supplementary Table 1:** Plasma Lipid Mediator Profiles

	Survivors				Non-survivors			
	Day 1		Day 5		Day 1		Day 5	
	Average	SEM	Average	SEM	Average	SEM	Average	SEM
DHA bioactive metabolome (*pg/mL*)								
RvD1	0.30	0.16	0.46	0.29	2.34	1.57	1.04	0.34
RvD2	0.68	0.20	2.98	2.16	0.63	0.29	13.46	7.22
RvD3	0.17	0.10	0.18	0.15	0.42	0.08	0.21	0.07
RvD4	0.98	0.55	1.31	0.63	3.81	1.34	2.24	0.94
RvD5	1.21	0.83	1.44	0.52	0.57	0.25	1.77	0.59
RvD6	0.72	0.40	0.73	0.25	0.54	0.17	0.40	0.22
17R-RvD1	1.00	0.81	0.11	0.06	0.21	0.17	0.38	0.15
17R-RvD3	0.12	0.05	0.10	0.09	0.08	0.06	0.26	0.20
PD1	0.84	0.46	4.74	3.98	0.80	0.52	3.39	2.05
10S,17SdiHDHA	5.94	4.21	9.35	8.39	7.75	4.49	3.60	1.78
17R-PD1	0.51	0.25	0.89	0.48	0.27	0.19	0.20	0.07
PCTR1	1.24	1.38	1.46	1.21	1.66	1.85	0.93	0.99
PCTR2	1.22	0.89	123.44	117.81	0.00	0.00	25.12	26.75
PCTR3	16.51	18.45	2.55	2.75	0.00	0.00	43.99	30.11
MaR1	0.00	0.00	1.44	1.01	0.00	0.00	3.81	1.65
MaR2	0.79	0.51	0.29	0.14	0.31	0.22	0.78	0.37
7S,14S-diHDHA	8.74	6.73	9.99	4.36	7.45	6.34	15.89	7.31
22-OH-MaR1	0.00	0.00	1.12	1.10	0.91	0.65	4.73	2.21
4S,14S-diHDHA	0.59	0.42	0.36	0.15	0.13	0.08	0.14	0.08
MCTR1	19.54	21.85	0.82	0.89	9.04	10.11	0.00	0.00
MCTR2	2.26	2.53	49.75	53.73	0.00	0.00	0.22	0.23
MCTR3	46.72	50.54	22.74	21.50	16.75	13.01	1.46	1.56
n-3 DPA bioactive metabolome (*pg/mL*)								
RvT1	0.38	0.38	0.67	0.41	0.35	0.25	1.55	0.88
RvT2	0.12	0.07	0.41	0.32	1.48	1.40	1.40	1.05
RvT3	0.48	0.14	5.06	5.14	0.73	0.31	1.00	0.55
RvT4	0.44	0.24	0.42	0.21	0.60	0.40	0.74	0.39
RvD1_n3 DPA_	0.77	0.28	0.48	0.09	0.47	0.19	1.81	0.61
RvD2_n3 DPA_	0.38	0.27	1.37	0.87	1.09	0.50	3.32	2.15
RvD5_n3 DPA_	1.05	0.55	1.49	0.79	1.18	0.88	1.21	0.96
PD1_n3 DPA_	0.81	0.47	0.37	0.17	1.22	0.23	0.30	0.22
10S,17S-diHDPA	0.60	0.30	0.12	0.13	0.62	0.43	0.48	0.47
PD2_n3 DPA_	0.26	0.20	0.41	0.18	0.39	0.35	0.06	0.06
MaR1_n3 DPA_	0.36	0.40	1.06	0.50	0.20	0.22	1.08	0.71
7S,14S-diHDPA	0.37	0.26	0.19	0.20	1.34	1.14	0.89	0.49
MaR2_n3 DPA_	5.58	1.92	5.36	1.90	6.62	3.72	8.67	5.95
EPA bioactive metabolome (*pg/mL*)								
RvE1	0.71	0.59	2.62	1.17	0.46	0.45	2.51	1.31
RvE2	0.46	0.36	0.59	0.28	0.00	0.00	3.36	3.30
RvE3	2.00	0.68	1.98	0.59	0.00	0.00	1.73	1.49
AA bioactive metabolome (*pg/mL*)								
LXA_4_	0.20	0.12	1.51	1.41	0.22	0.17	0.35	0.22
LXB_4_	56.89	33.64	28.43	9.18	49.68	26.23	32.28	14.62
5S,15S-diHETE	0.67	0.75	0.70	0.55	0.94	0.51	0.46	0.24
15-epi-LXA_4_	0.76	0.46	2.25	1.24	4.03	2.48	4.85	2.73
15-epi-LXB_4_	0.00	0.00	5.99	3.66	0.93	0.80	1.39	1.04
LTB_4_	59.61	39.43	19.08	10.53	13.32	7.91	7.38	2.79
5S,12S-diHETE	0.57	0.64	0.85	0.44	2.51	1.95	3.91	2.56
20-OH-LTB_4_	39.32	24.93	41.82	25.07	7.22	4.76	19.70	15.52
LTC_4_	104.49	42.32	245.07	58.23	140.27	60.57	380.26	212.29
LTD_4_	2.28	1.08	2.57	2.48	2.46	2.32	5.54	3.88
LTE_4_	99.42	75.69	44.22	22.49	17.65	5.42	15.99	2.57
PGD_2_	47.06	32.13	13.55	5.12	26.30	15.98	10.14	4.67
PGE_2_	158.05	127.58	49.01	23.36	62.15	60.14	11.75	2.49
PGF_2a_	92.10	66.27	32.92	16.63	129.55	135.36	15.65	4.06
TXB_2_	760.53	562.20	280.12	158.57	441.14	291.99	761.01	632.45

AA, arachidonic acid; DHA, docosahexaenoic acid; DPA, docosapentaenoic acid; EPA, eicosapentaenoic acid; SEM, standard error of the mean.

**Supplementary Table 2:** Plasma Cytokine and Chemokine Levels

Plasma analyte	Survivors				Non-survivors				HV	
	Day 1		Day 5		Day 1		Day 5			
	pg/mL	SD	pg/mL	SD	pg/mL	SD	pg/mL	SD	pg/mL	SD
CCL2	378.4	174.6	323.7	131.1	266.1	192.9	319.4	194.3	159.4	51.9
CCL3	41.8	17.8	31.6	46.4	38.9	16.4	27.4	12.9	13.9	2.8
CCL4	159	114.1	135.1	100.8	99.5	19.8	92.1	27.6	34.9	5.8
CCL17	80.3	12.2	126	34.2	157.3	30.5	86.9	17.1	50.3	21.3
CCL22	378.9	74	764.5	164.6	932	136.8	408	62.4	838.7	200.3
Eotaxin	914.8	480.8	885.1	315.4	842.2	459.4	1047.8	429.6	647.4	126.4
Eotaxin-3	138.5	66.1	150.7	59.3	117.4	43.2	142.7	60.7	647.4	126.4
GM-CSF	0.18	0.04	0.17	0.04	0.18	0.04	0.10	0.03	0.06	0.03
IFN-γ	8.54	2.53	9.14	2.18	3.29	1.05	6.16	2.24	1.78	0.99
IL1α	2.05	3.21	2.67	1.82	4.85	5.2	4.52	3.70	n/a	n/a
IL1β	0.59	0.14	1.7	0.42	0.41	0.12	1.34	0.95	n/a	n/a
IL2	0.62	0.27	0.89	0.35	0.36	0.23	0.58	0.33	1.17	1.06
IL4	0.082	0.02	0.19	0.05	0.065	0.02	0.056	0.01	0.03	0.01
IL5	0.73	0.5	1.17	1	0.67	0.26	1.04	0.74	0.56	0.20
IL6	17.5	7.16	20.68	5.08	26.3	9.66	20.94	9.5	0.13	0.04
IL7	6.43	1.49	4.95	0.84	5.20	1.07	5.32	0.91	2.93	0.63
IL8	265.4	35	359.3	202.4	261.1	68.7	156.8	34.9	3.6	1.14
IL10	1.06	0.24	1.03	0.34	1.13	0.28	1.63	0.43	0.13	0.07
IL12p70	0.45	0.37	0.58	0.60	0.29	0.07	0.58	0.60	0.18	0.07
IL13	2.12	1.53	2.82	2.13	1.59	0.85	2.19	2.85	0.72	0.39
IL15	7.28	1.7	5.73	1.9	7.36	4.13	6.0	1.58	2.68	0.51
IL16	358.9	218.6	341.3	250.2	472.3	543.05	511.2	344.1	142.8	35.17
IL17	5.97	1.86	6.01	1.78	3.26	0.39	2.59	0.28	2.18	0.74
IL23	99.23	124.6	90.1	61.54	94.68	79.16	64.9	54.9	105.7	52.3
IP-10	1104.1	1040.9	1085.2	568.5	729.3	499.3	1085.2	568.5	220.4	63.3
MCP-4	334.5	81.8	443.4	51	344.3	45.6	636.6	80.9	243.4	96.8
TNF-α	4.45	0.83	4.05	0.97	5.52	1.44	4.33	0.78	1.26	0.11
TNF-β	0.53	0.17	0.42	0.10	0.52	0.14	0.48	0.11	0.29	0.06
VEGF	130.9	163.3	125.7	157.1	32.71	20	103.4	172.8	12.38	2.04

GM-CSF, granulocyte-macrophage colony-stimulating factor; IFN, interferon; IL, interleukin; IP-10, interferon gamma–induced protein 10; MCP, monocyte chemoattractant protein-4; SD, standard deviation; TNF, tumor necrosis factor; VEGF, vascular endothelial growth factor.

## Supplementary Methods

### Criteria for Decompensation of Liver Cirrhosis

These were defined as acute onset or worsening of complications of cirrhosis including gastroesophageal hemorrhage, jaundice, alcoholic hepatitis, ascites, hepatic encephalopathy, sepsis, or infection including spontaneous bacterial peritonitis, hepatorenal syndrome, or renal dysfunction.

Renal dysfunction was defined as an increase in serum creatinine level by ≥50% compared with creatinine level at baseline. Infection was defined as when a patient was prescribed a new antibiotic(s), and clinical data were recorded to subsequently categorize infection according to standard international criteria.^1^ Both of these endpoints were recorded no earlier than 48 hours after the onset of albumin treatment.

### Monocyte-Derived Macrophages Ex Vivo Immune Function Assay

MDMs obtained from healthy volunteers^2^^,^^3^ were sensitized with plasma for 30 minutes at 37°C and then stimulated with 100 ng/mL LPS (*Salmonella abortus equi* S-form, TLRgrade; Enzo Life Sciences, Exeter, United Kingdom). Cell supernatants were collected at 4 and 24 hours after stimulation and stored at –80°C. TNF-α (4-hour samples) and IL10 levels (24-hour samples) in supernatants were quantified by enzyme-linked immunosorbent assay (DuoSet ELISA; R&D Systems, Minneapolis, MN) following manufacturer’s protocol. For selected experiments pan-PGE_2_ receptors were antagonized with 50 μmol/L AH6809 (EP1-3 antagonist) (Cayman Chemicals, Ann Arbor, MI) and 10 μmol/L MF498 (EP4 antagonist) (Cayman Chemicals) before plasma sensitization and LPS stimulation.

For soluble IL4 blocking experiments, plasma from healthy volunteers or ATTIRE patients was incubated with either 1 μg/mL human IL4 antibody (clone #34019; R&D Systems) or mouse immunoglobulin G_2B_ isotype control (clone #20116; R&D Systems) for 1 hour at 37°C before MDM sensitization. In other experiments recombinant human IL4 (PeproTech, Rocky Hill, NJ) at different concentrations were added to healthy volunteer plasma.

### Plasma Soluble Mediator Analysis

Circulating albumin levels were measured at local hospital laboratories as part of the study protocol. Plasma LBP levels were quantified by ELISA following manufacturer’s protocol (R&D Systems). Briefly, plasma was thoroughly thawed and diluted in reagent diluent (phosphate-buffered saline containing 5% bovine serum albumin) before being added to antibody-coated microplates. Quantification of immobilized antibody-enzyme conjugates was performed by monitoring horseradish peroxidase activities in the presence of the substrate 3, 3′, 5, 5′-tetramethylbenzidine, which was measured spectrophotometrically by the increased absorbency at 450 nm.

Levels of multiple cytokine and chemokine levels were measured in patient and healthy volunteer plasma by electrochemiluminescence detection using the *V-Plex* Pro-inflammatory panel 1, Cytokine Panel 1, and Chemokine Panel 1 kits from Meso Scale Discovery (Rockville, MD). Data were acquired by using a SECTOR S 6000 plate reader (Meso Scale Discovery).

Plasma LM levels were quantified by high-performance liquid chromatography as described.^3^

### Western Blotting

PBMCs were obtained from acutely decompensated inpatients admitted to University College Hospital with complications of cirrhosis or healthy volunteers. Full National Health Service Research Ethics Committee ethical approval was obtained (REC Reference number 15/LO/0800).

Briefly, venous blood was collected in vacutainer tubes containing heparin and layered onto 15 mL Ficoll-Paque PLUS (GE Healthcare, Little Chalfont, United Kingdom). PBMCs were separated by density gradient centrifugation, and residual erythrocyte contamination was removed by using ammonium-chloride-potassium lysis buffer (Lonza, Basel, Switzerland). Cell pellets were then lysed in RIPA buffer (Sigma-Aldrich, St Louis, MO) supplemented with protease inhibitors. Thirty micrograms of cell lysates was then loaded onto a 4-15% Tris-Glycine gel (Bio-Rad, Hercules, CA), electrophoresed, and transferred onto a polyvinylidene difluoride membrane. Membrane was immunoblotted with rabbit monoclonal anti-IL4 antibody (ab62351; Abcam, Cambridge, United Kingdom) and mouse monoclonal anti-GAPDH (sc-32233; Santa Cruz Biotechnology, Dallas, TX). Anti-rabbit (sc-2301; Santa Cruz Biotechnology) or anti-mouse (NA931VS, GE Healthcare) horseradish peroxidase–tagged antibodies were used for secondary binding, and chemiluminescence (Clarity Western ECL; Bio-Rad) was used to visualize proteins.

### Statistical Analysis

Unless stated otherwise, data are presented as mean ± standard deviation. Two-tailed (unpaired) *t* tests were performed when comparing 2 independent groups of values where a normal distribution was reasonable to assume.
